# Social Media for Health Campaign and Solidarity Among Chinese Fandom Publics During the COVID-19 Pandemic

**DOI:** 10.3389/fpsyg.2021.824377

**Published:** 2022-01-19

**Authors:** Qiaolei Jiang, Shiyu Liu, Yue Hu, Jing Xu

**Affiliations:** ^1^School of Journalism and Communication, Tsinghua University, Beijing, China; ^2^School of Journalism and Communication, Peking University, Beijing, China; ^3^Department of Political Science, Tsinghua University, Beijing, China

**Keywords:** affordance, social media, public health, solidarity, COVID-19 pandemic, face mask, fandom publics

## Abstract

**Background:**

As a highly contagious disease, the COVID-19 pandemic has become a serious health threat and psychological burden for the global communities. From the conceptual perspective of affordances, this research examined the role of social media for health campaign and psychological support during the global crisis.

**Methods:**

Data from both social media and a nationwide survey were collected and analyzed. Face mask-related posts on Sina Weibo from January 1, 2020, to June 30, 2020, were retrieved and studied. Face mask wearing as a well-established preventive measure was identified and examined from hashtag topics. A nationwide survey with a randomized experiment embedded was conducted to further investigate the mobilizing dynamics.

**Results:**

During the escalation phase in the first half of 2020, the hashtag topic “#national mask campaign#,” initiated by a celebrity, topped the ranking of mask-related topics on Weibo. The findings indicated that prevention activities, solidarity expression, and names of celebrity idols were frequently discussed. With celebrity idols as opinion leaders, millions of fandom publics actively participated in this hashtag health campaign. Results of the nationwide survey show the popularity of fan identity, and the experiment results demonstrate the strong mobilizing power of celebrity idol and fandom community in civic engagement and participation among young Chinese.

**Conclusions:**

The research demonstrates how the affordances of social media, such as liking, commenting, reposting, and hashtagging can be influential in promoting health behaviors and expressing solidarity. Implications for public health professionals and policymakers to raise awareness and understanding about health campaigns *via* social media are discussed.

## Introduction

The COVID-19 pandemic has become the most extensive affliction to humanity globally since its outbreak in December 2019. The WHO declared the novel coronavirus outbreak as a public health emergency of international concern on January 31, 2020, and declared the COVID-19 outbreak as a global pandemic on March 11, 2020 (WHO, 2020a). According to the Johns Hopkins Coronavirus Resource Center (JHCRC), by September 23, 2021, there have been over 230 million confirmed cases and roughly 4.7 million deaths caused by the COVID-19 worldwide (Johns Hopkins Coronavirus Resource Center, 2021). As a crisis for the entire world and a daunting challenge, it has not only posed a grave threat to human life and health but also brought unbearable psychological pressure (Cao et al., 2020).

The best way to prevent COVID-19 is to avoid being exposed to the deadly virus. In the context of the global COVID-19 pandemic, face mask wearing has become usual and ubiquitous (Lepelletier et al., 2020). However, in the early stages of the COVID-19 pandemic, it took time and great effort to encourage and convince people to wear masks to prevent the spread of COVID-19.

In the early stage of the COVID-19 outbreak, there was no validated treatment for COVID-19. Global, national, and local health organizations issued guidelines and safety measures to protect people from the deadly virus. Since late January 2020, the WHO has released a series of documents to give advice on the use of masks during the COVID-19 pandemic (WHO, 2020b). Updated documents aiming at minimizing the spread of COVID-19, available in 26 languages, suggest that as part of a comprehensive package of the prevention and control measures during the pandemic, public using face masks and keeping social distance in the community can reduce COVID-19 transmission (WHO, 2020b). Empirical results provide evidence that mask wearing can help to prevent transmission of SARS-CoV-2 (Brooks et al., 2020; Leung et al., 2020). Centers for Disease Control and Prevention in both China and the US call on people to wear mask to prevent COVID-19 spread (The National Bureau of Disease Control Prevention of China, 2020; U.S. Centers for Disease Control Prevention, 2020).

However, there are still many concerns regarding mask wearing, due to multiple factors, from inconvenience to rumors such as face masks with 5G antennas and 5G signals which can control people wearing masks and give them cancer (Sbraccia, 2020), and even political issues in some social contexts (Kahane, 2021). There are still antimask attitudes and even antimask protests that happened in some places after the implementation of mask-wearing mandates, ordinances, or laws.

Therefore, to wear a mask or not to wear a mask, that is an important question during the COVID-19 pandemic, especially with the resurging of COVID-19 and multiple variants emerging around the world. During a special time of social distancing, quarantine, and isolation due to the global COVID-19 pandemic, individuals are more likely to rely on media when they do not have first-hand experience or knowledge. In previous studies, mass media have long been regarded as important shapers of people's perceptions (Coleman, 1993; Morton and Duck, 2001; Chang, 2012; Oh et al., 2015). With the affordances of digital technologies transforming people's media experience (Sundar, 2008; Sundar and Limperos, 2013), digital health interventions can play a more influential role during an infectious disease outbreak (Mohr et al., 2011; Kim et al., 2016). However, current empirical studies are far from enough to answer the questions, such as what are the specific possibilities for action provided by digital media for people, and how purposeful action and shared understanding are situated within a certain context. To fill the gap, based on data collected in the first half of 2020 in China when it was during the early stage of the COVID-19 outbreak, this research investigates face mask-wearing issue from the conceptual framework of affordances, so as to showcase the role of social media for health campaigns and psychological support during the COVID-19 pandemic and explore the mechanism of identity processes in collective action *via* social media.

## Literature Review

### Affordances: A Conceptual Framework

Rooted in the field of ecological psychology, affordances as a concept were first coined by Gibson (1979), which means possibilities for action provided by the environment. The term affordances have been widely applied in communication studies, which closely link the concept with media technologies as dynamic, emerging from the relationship between the user and specific technologies (Evans et al., 2017). The perspective of affordances offers a relational view to explain media use at different levels (Leonardi and Barley, 2008).

There have been emergent trends of affordance research focused on new media and communication (Rietveld and Kiverstein, 2014; Evans et al., 2017). Abundantly used, social media have the capabilities of allowing people to be connected and exchange information in a variety of formats, which has a profound impact on people's lives. Compared with traditional mass media, such as newspapers, radio, and TV, social media empower users to see how people are connected with each other, how people are connected to content, and how content is connected (Treem and Leonardi, 2012). Therefore, organizing processes, such as communication, collaboration, and knowledge sharing, can be structured by social media affordances (Leonardi and Vaast, 2017).

The affordances of social media make it possible for users to communicate with each other, promote collaboration in a wide range of contexts, and get involved in knowledge sharing in different ways (Majchrzak et al., 2013). For example, affordances of metavoicing are common and popular since the advent of social media because users can both share their ideas and receive reactions (Majchrzak et al., 2013). Metavoicing has abundant forms, including retweeting, commenting, liking, and voting, which make the online discussion more interactive and productive. The narrative agency may lead to “groupthink,” which may not seem to be particularly positive but is the basis of collective action (Janis, 1972; Yang, 2016). Triggered attending, as another typical social media affordance, can alert users that any changes are made to the content they focus on (Majchrzak et al., 2013). For example, the users can be notified when the pages or messages they are monitoring have been updated. They can choose to become engaged or not. There are many challenges to attracting and retaining engaged individuals for online communities (Ma and Agarwal, 2007), while triggered attending can be a way to promote engagement by informing users that the social media platforms care about the followers. In this way, triggered attending provides individuals with an efficient way to participate in online discussions and encourages followers to pay attention to the ongoing collective discussion.

As one of the emergent social media modalities, hashtags have an increasing presence in people's daily life for civic purposes, especially among the young (Mihailidis, 2020). Since the first hashtag tweet was posted on Twitter in 2007 (Kirkpatrick, 2011), hashtags have become popular and have been used widely and regularly by millions of social media users, especially among users of microblogging platforms. Hashtags refer to the use of short words or phrases that follow the hash or pound sign (#) on social media platforms. As an innovative tool, hashtags make it possible for those who are not connected to a user to see and comment on the messages (Bruns and Burgess, 2011).

Hashtagging has become so powerful because it contains community elements to increase engagement. The most typical example is the “hashtag movement,” which has been popular worldwide in recent years. This type of movement is possible due to the affordances of social media platforms. Online topics are aggregated through unified labels and can be transferred to the offline world after accumulating enough discourse power. For example, the widely known #MeToo, #IceBucketChallenge, and #BlackLivesMatter movements are familiar to the global public. In these large-scale social movements, people participated extensively and deeply. Meanwhile, because the tags inspire and even construct translocal communication, which can be radicalized under certain social circumstances and transformed into collective identity through sharing and transmitting common emotional experiences.

In light of characteristics of social media and online behaviors, affordances as a conceptual framework provide an effective lens that can help us to understand how people share information, get involved in public issues, and become engaged in collective action online and offline. During the COVID-19 pandemic, when social distancing, quarantine, and isolation have become daily experiences, social media act as a considerable channel for people to receive health information and empower the public to foster online discussion. Therefore, this research attempts to investigate how preventive interventions such as mask wearing were discussed on social media in China during the pandemic and also the role and impact of social media affordances for health campaigns and psychological support. Hence, the first research question was proposed.

RQ1: How face mask was represented on China's twitter-like social media Sina Weibo during the COVID-19 pandemic?

### Computer-Mediated Communication (CMC), Identity, and Collective Action

There are continuous debates on the impact of hashtag activism online vs. real world action-taking (Bonilla and Rosa, 2015; Mihailidis, 2020); hence, it is important to investigate the mechanism of collective action *via* CMC.

In a broad definition, CMC can be defined as human communication *via* all kinds of digital technologies, such as various social media, which channel and shape communication and social behaviors (Herring, 2004; Priante et al., 2018). The dramatic CMC has been brought out by the distribution of cheap computers since around the 1990s. People with different cultures, languages, backgrounds, and work practices are connected by CMC, which presents a deep influence on interpersonal interaction, communication efficiency, information exchange, and social behaviors (Haythornthwaite, 2005). CMC has the potential to break down the boundaries, such as nationality, race, language, and social status and can create a conducive platform for public deliberation by avoiding undesirable social-psychological influences, such as fear of isolation and communication apprehension (Postmes et al., 1998). Therefore, CMC gives people more freedom to express themselves and contact other social groups in online communities.

There are two forms of collective action *via* CMC, i.e., CMC-based collective action which takes place online and exists only because of CMC and CMC-supported collective action which can be traditional collective action happening offline with CMC being used as a channel to organize and communicate (Priante et al., 2018). Although the importance of identity has been widely recognized in the research of collective action *via* CMC, the role of identity has become a source of contention (Earl and Kimport, 2011; Earl et al., 2014; Bakardjieva, 2015; Gerbaudo and Treré, 2015; Priante et al., 2018). To examine the role of identity in collective action *via* CMC, either CMC-based or CMC-supported can advance our understanding of the coherence, organization, and mobilization of collective action (Bakardjieva, 2015; Gerbaudo and Treré, 2015; Priante et al., 2018).

Identity process in collective action, including the construction, maintenance, and negotiation, has been greatly changed by CMC (Russell, 2005; Wall, 2007; Stein, 2009; Earl and Kimport, 2011; Earl et al., 2014; Milan, 2015; Priante et al., 2018). For example, social media provide means and spaces for easily, quickly, and even creatively expressing, constructing, sharing, and negotiating identities which also become symbols of campaigns and movements, including digital health interventions (Mohr et al., 2011; Kim et al., 2016; Priante et al., 2018). Thus, it is important and necessary to understand how social media affordances affect identification practices in collective action. The lens of affordances can be helpful for exploring why, how, and when new technologies, such as social media, become enrolled in and affect collective action (Majchrzak et al., 2013).

Collective identity highlights we-ness and collective agency (Snow, 2001), which motivates people to be part of a group, and fosters collective action. The use of social media has played an instrumental role in the identity process during collective action. On social media, such as Facebook, Twitter, and Sina Weibo in China, people use text, icons, symbols, and images to foster identity development. The affordances of CMC, especially social media, are an important driving force for the formation of mediated publics, networked publics, affective publics, issue publics, and fandom publics (Papacharissi, 2014; Zhang, 2016). The anonymity of social media may lessen the hesitancy of members who hold different viewpoints and lead to more communicative discussions, in which people are assembled to participate in online discussions and collective action.

As a specific social category, fans are considered to have social connections and participation involved in politics and other public issues (Fiske, 1992). Fan activities are based on communities, where they invest intense passion in the activities they involve, participate in strong communal discussions of topics, and engage in civic-oriented activities. Categorizing themselves as a community makes solidarity among strangers possible. Social media, excellent sites for fan activities, have rapidly increased the speed and intimacy of virtual interactions among fans, making it possible to create and sustain online communities for fans, where they collaborate on collecting information, sharing experiences, spreading news, expressing emotional reactions, and even planning mobilization tactics together. With emotional mobilization, media technologies, such as social media, as the foundation of a network society (Castells, 2006), turn fans into fandom publics (Zhang, 2016). With people, especially the young, immersed in digital culture and booming fandom culture, this research pays special attention to fandom publics, with a focus on CMC's impact on their identity development and the role of identity in collective action.

Driven by the contention over the role of identity in collective action *via* CMC in existing studies, the mechanism of social media health campaign during the COVID-19 pandemic is also explored in this research by examining the interrelationships among CMC, identity, and collective action. During the pandemic as a global crisis, people get engaged with content on social media in their personal ways that they not only act but actively construct meaning (Sundar, 2008; Sundar and Limperos, 2013). Regarding social media as prospective tools for public health campaigns, this research aims to explore the role of identities, such as fan identity and fandom identification, in collective engagement and participation online and even offline during the pandemic. Therefore, the following research questions were proposed.

RQ2: How popular are fan identity and fandom identification among young Chinese? What are the roles of celebrity idol and fandom publics in collective engagement and participation during the COVID-19 pandemic?

## Method Study 1

### Data Source and Collection Procedure

For Study 1, one of the most widely used social media, Sina Weibo, a twitter-like microblog platform was used for data collection and quantitative analyses. By adopting a keyword maximization search method, this study used “mask, face mask, N95, N90, N100, 3M, DS2, KN95, KN90, FFP1, FFP2, FFP3, and KF94” in Chinese as keywords to obtain posts on Sina Weibo from January 1, 2020, to June 30, 2020. The data collected using the abovementioned search strategy accumulated 88,078,456 posts, including both original and forwarding mask-related Weibo posts, in the first half of 2020 during the COVID-19 pandemic.

### Topic Identification

On Sina Weibo, certain content that is preceded by a hashtag sign (#) is regarded as a specific topic. Therefore, topics related to face mask were identified, and the ranking of the topics was generated according to the amount of Weibo users' reposting, commenting, and liking.

### Text Data Preprocessing

Data cleaning and word segmentation as the preprocessing procedure were conducted for the obtained text data of Weibo posts. Specifically, Python (Python Software Foundation) was used to remove error information effectively. After data cleaning, the Python package Jieba was used for word segmentation, which converted sentences into words for further analyses.

### Social Media Profiling

To better understand the sources of the posts and those who were engaging, the social profile attributes from the Weibo data as individual and group profiling, such as age, gender, location, and user interests, were inferred. The spider, an automated web crawler, and the company's application program interface were used to extract data from users' profile information.

## Results Study 1

### Face Mask Became a Hot Word in the Early Days of the COVID-19 Pandemic

**Figure 1:**
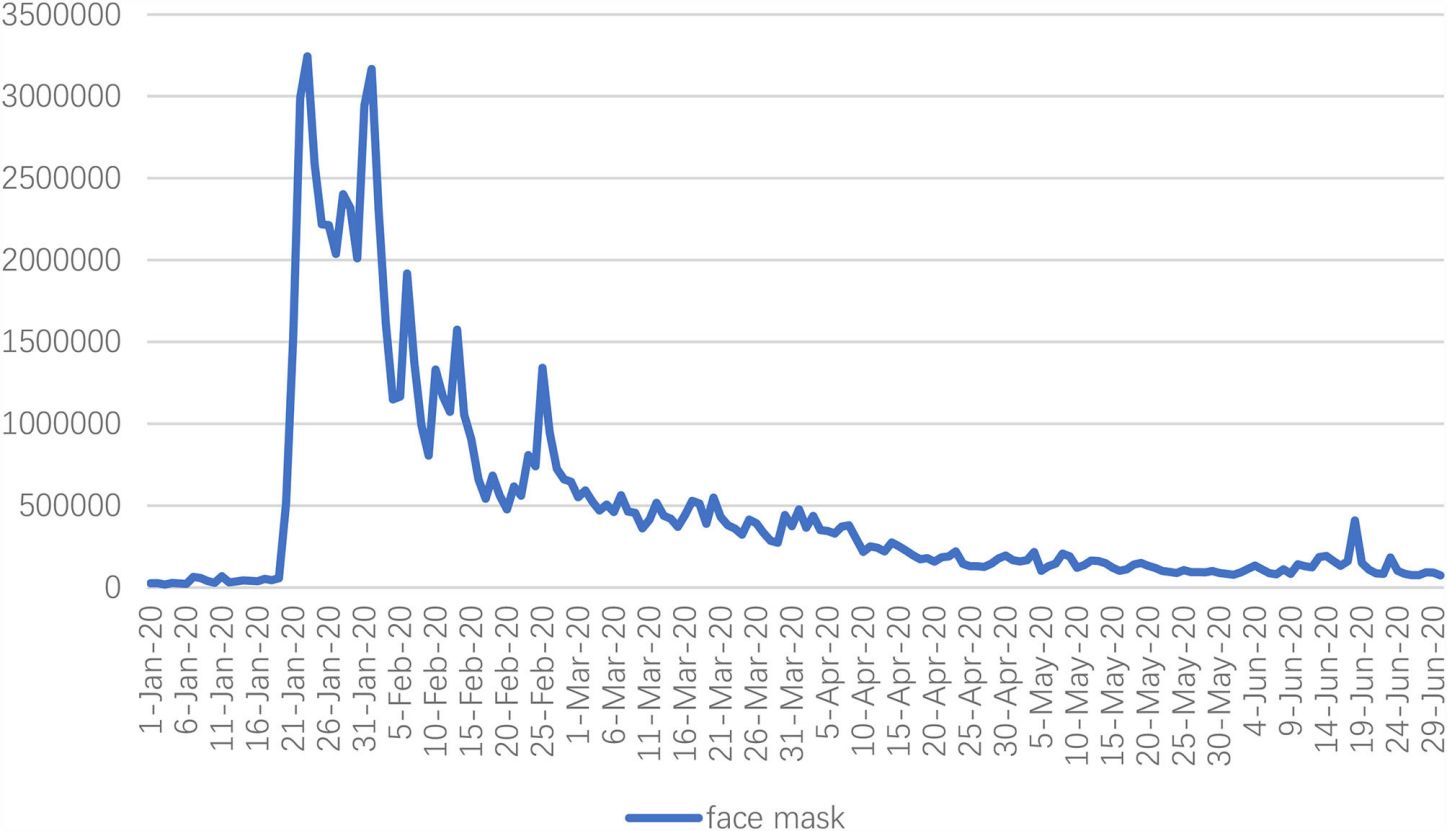
Face mask mentions on Sina Weibo.

Based on the obtained Weibo data, this study first attempted to explore the public awareness of mask wearing on Sina Weibo by examining the frequency of “face mask” occurrence. According to the results shown in Figure 1, the trend of “face mask” occurrence on Sina Weibo has changed in the early days of the COVID-19 pandemic. The face mask mentions peaked at 3,243,705 on January 23, 2020, right after the official media interview with China's top epidemiologist Dr. Nanshan Zhong, who mentioned person-to-person transmission of the novel coronavirus.

### Hashtags in Weibo Mask-Related Health Campaign

To examine the content of public discussion about face mask on Sina Weibo, Weibo hot topics were identified and investigated. As Twitter, adding hashtags can identify certain content as concerning a specific topic. The findings show that “#national mask campaign#” topped the Weibo hot topic ranking with an accumulated amount of 7,552,804 topics being discussed about face mask by June 30, 2020. As the top 1 hot topic, “#national mask campaign#” constituted 25.81% of the total discussion among the top 1,000 hot topics of face mask on Sina Weibo. Therefore, the hot topic “#national mask campaign#” was selected as a case to further investigate the social media health campaign on Sina Weibo.

### “#National Mask Campaign#” on Sina Weibo

**Figure 2:**
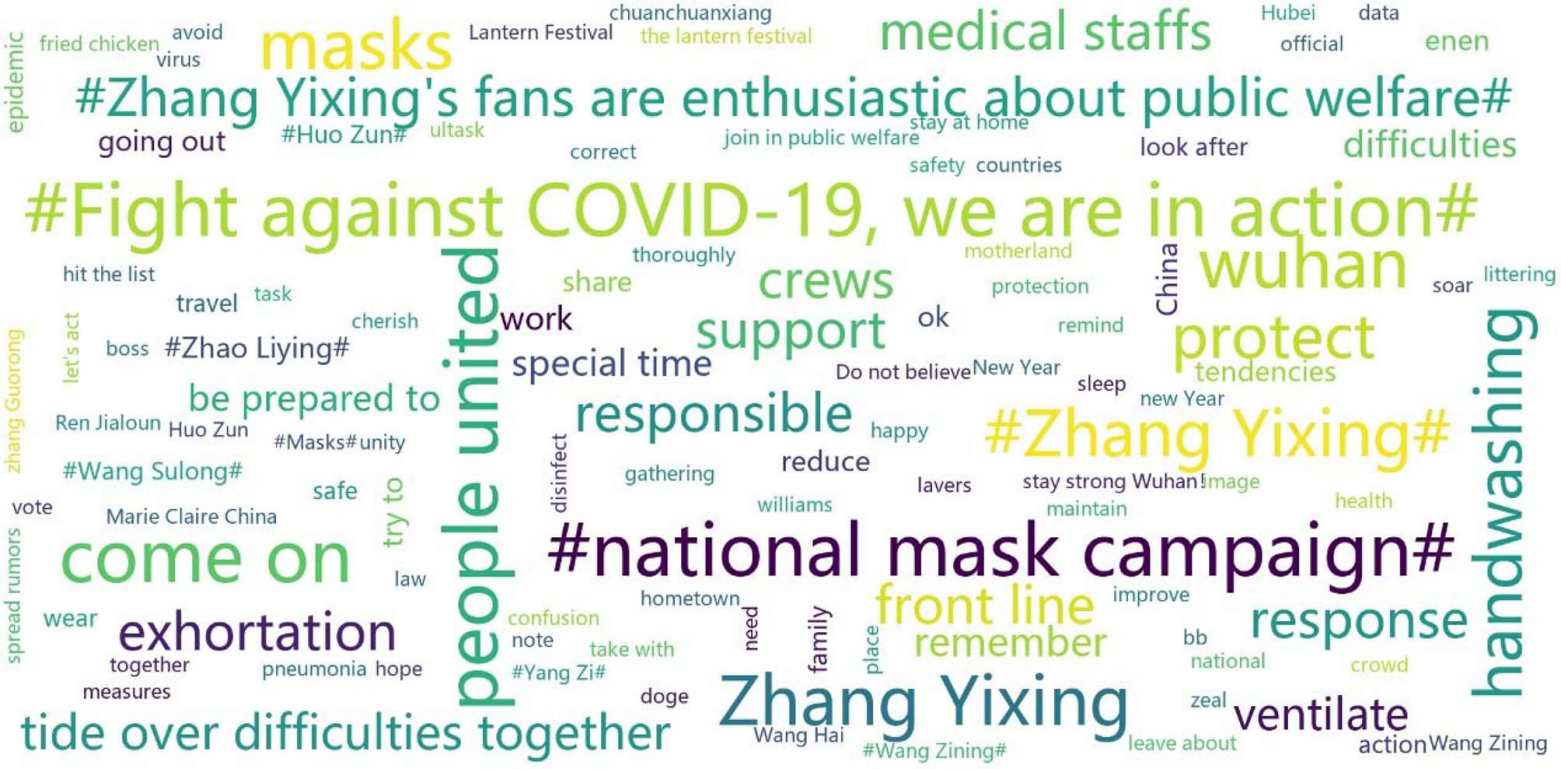
Word cloud of “#national mask campaign#” on Sina Weibo.

To identify and visualize those high-frequency words or phrases in the hot topic of “#national mask campaign#” on Sina Weibo, word cloud analysis was adopted. Figure 2 further demonstrates the content of this social media health campaign. The most frequently appeared words show the impact of hashtags for expressing affiliation or interests in the topics, such as “#national mask campaign#,” “#fight against COVID-19, we are in action#,” and “#Zhang Yixing's fans are enthusiastic about public welfare#.” Together with “masks” being regarded as important prevention of COVID-19, other preventions, such as “handwashing,” “medical staffs,” “ventilate,” and “stay at home,” were also highly discussed. Solidarity was also widely emphasized within the social media health campaign, such as “come on,” “people united,” “tide over difficulties together,” “protect,” “support,” “Wuhan,” and “front line.” Meanwhile, it is worth mentioning that there were many names of celebrities being highly appeared, for example, “Zhang Yixing,” “Zhao Liying,” “Wang Zining,” “Huo Zun,” “Wang Sulong,” and “Yang Zi,” indicating celebrities and their fans' active participation in the Weibo “national mask campaign.”

To further examine the “#national mask campaign#,” all the related hashtag posts were analyzed and sorted based on the frequency of liking, commenting, and sharing on Weibo. The first hashtag “#national mask campaign#” was posted by a verified Weibo account Xie Binbin, who is an actor and singer. Therefore, the “#national mask campaign#” on Weibo was initiated by a Chinese celebrity. The results show that those most influential posts were also posted by those verified celebrity Weibo accounts, including Dilraba, Zhang Yixing, Yang Mi, Meng Meiqi, Deng Lun, Ren Jialun, Yang Zi, and Angelababy, who are all Chinese pop stars regarded as celebrity idols by the young in China. With millions of views and reposts, hundreds of thousands of comments and likes, these famous celebrities' Sina Weibo posts raised the considerable concern of mask wearing as one of the most effective public health measures and part of a comprehensive strategy of measures to prevent the spread of COVID-19.

To explore the role and influence of these celebrities' posts, the post with the most reposts was chosen for further analysis. The hashtag post “#national mask campaign#” with most reposts was posted by Dilraba, a Chinese actress and singer, who is also a very popular celebrity idol in China. This post was reposted more than one million times, with 7,65,586 comments, 4,33,046 likes, and 2.13 million views. The results in Table 1 show how Dilraba's “#national mask campaign#” post got spread through eight levels of reposting on Sina Weibo. Based on the number of reposts, reposting accounts, and the related proportion, the results indicate that the third and fourth levels of reposting were more influential, with 3,36,249 reposts (51.96%) by 1,37,153 reposting accounts and 6,41,703 reposts (36.03%) by 95,608 reposting accounts, respectively.

**Table 1:** Spreading of the most influential “#national mask campaign#” post by levels of reposting on Sina Weibo.

**Level of reposting**	**Number of repost(s)**	**Number of reposting accounts**	**Proportion of reposting**	**Number of repost(s) of the top 5 reposting accounts in each level**
1	37,832	22,990	8.66%	10,953
				10,699
				7,730
				7,131
				5,748
2	53,090	11,161	4.21%	95,401
				34,656
				22,028
				15,479
				14,851
3	3,36,249	1,37,153	51.69%	250,102
				41,741
				37,860
				33,291
				30,043
4	6,41,703	95,608	36.03%	239
				223
				159
				159
				158
5	1,339	323	0.12%	2
				1
6	4	3	0%	1
7	1	1	0%	1
8	1	1	0%	0

**Figure 3:**
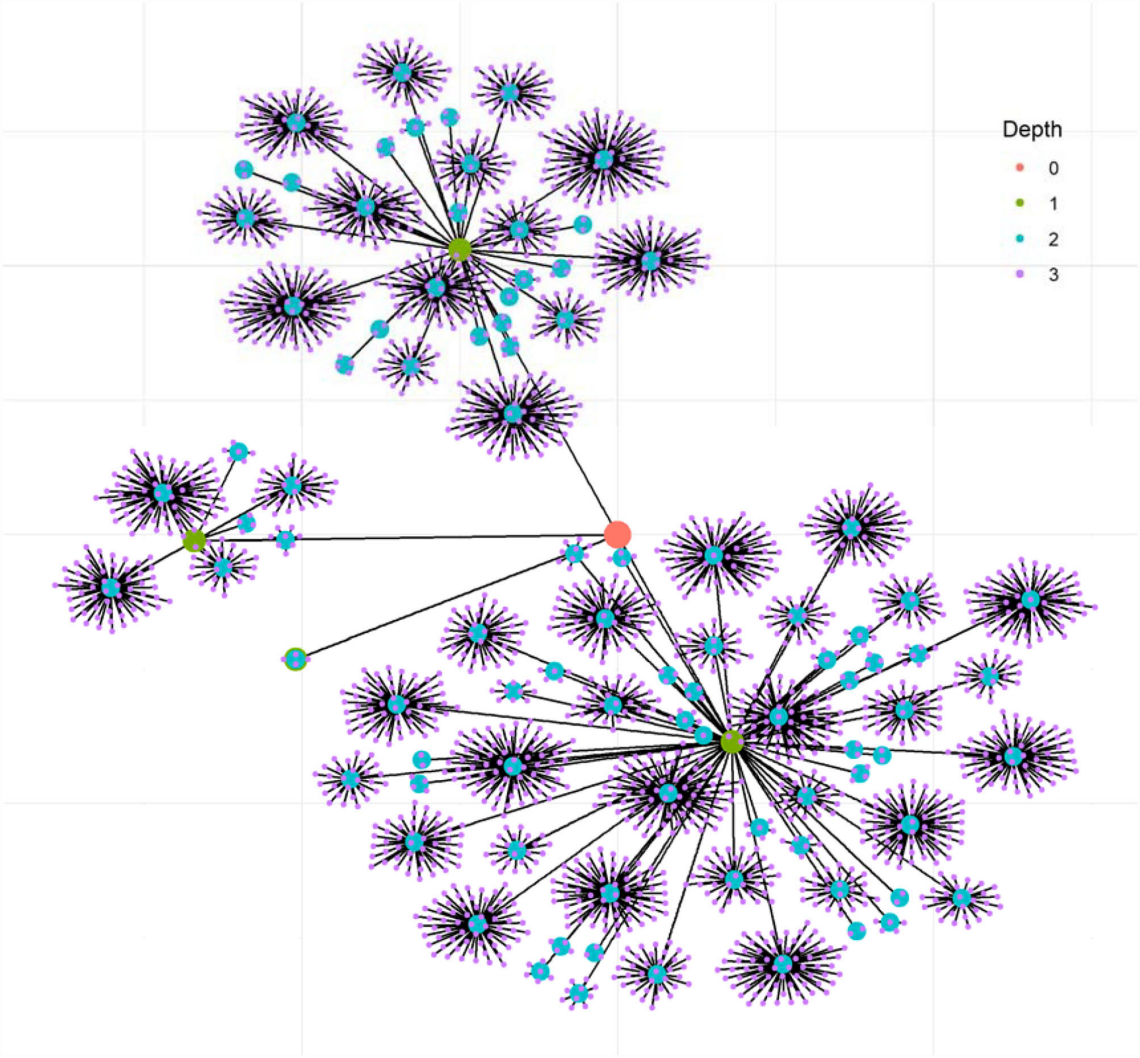
Illustration of the reposting of Diliraba's “#national mask campaign#” on Sina Weibo. This plot illustrates how Diliraba's “#national mask campaign#” is being spread through levels of reposts on Sina Weibo. The network was simulated based on 1:10,000 statistics of the real data.

To better illustrate the spread of the most influential “#national mask campaign#” post on Sina Weibo, the visulization of a random network simulation was generated by ggraph version 2.0.5 in R (version r80314) based on the 1:10,000 ratio of the real data. Figure 3 shows the spreading paths with the four inner levels of the reposting because the four outer levels accounted for only a tiny percentage of the reposting (refer to Table 1). The red dot in the center represents the initial “#national mask campaign#” post by Dilraba. The second-, third-, and fourth-level of reposts were represented by green, blue, and pink dots. The edges represent the reposting paths. Figure 3 shows a clear hierarchical spreading network from the celebrity idol to fandom influencers and fandom publics.

### Hashtag Participation and Fandom Publics

**Figure 4:**
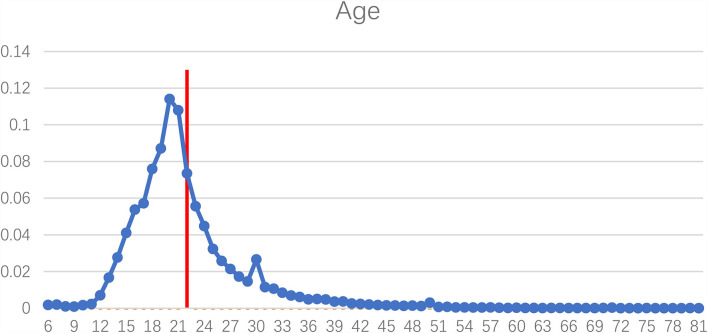
Age distribution of the hashtag “#national mask campaign#” participants.

**Figure 5:**
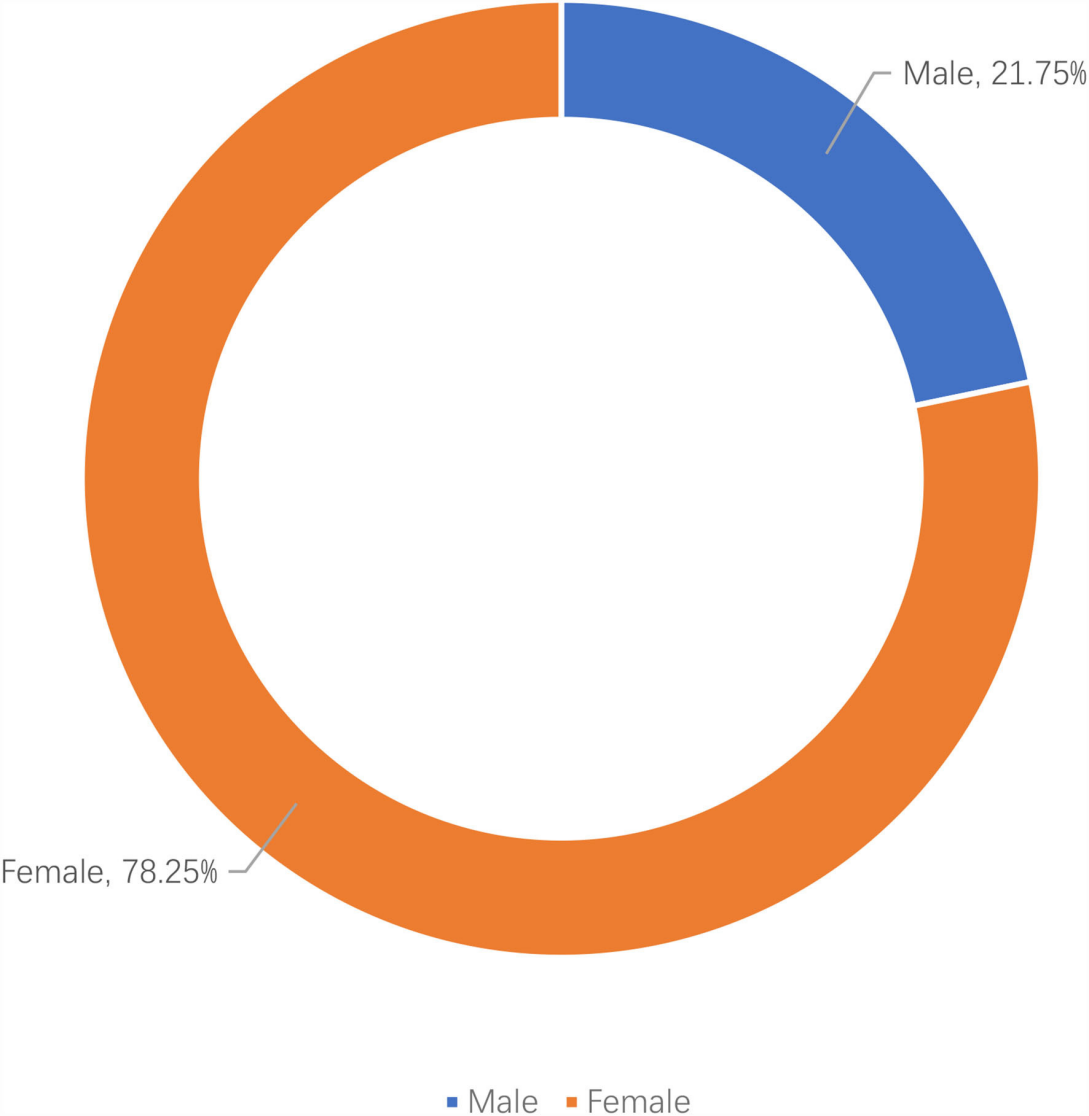
Gender distribution of the hashtag “#national mask campaign#” participants.

**Figure 6:**
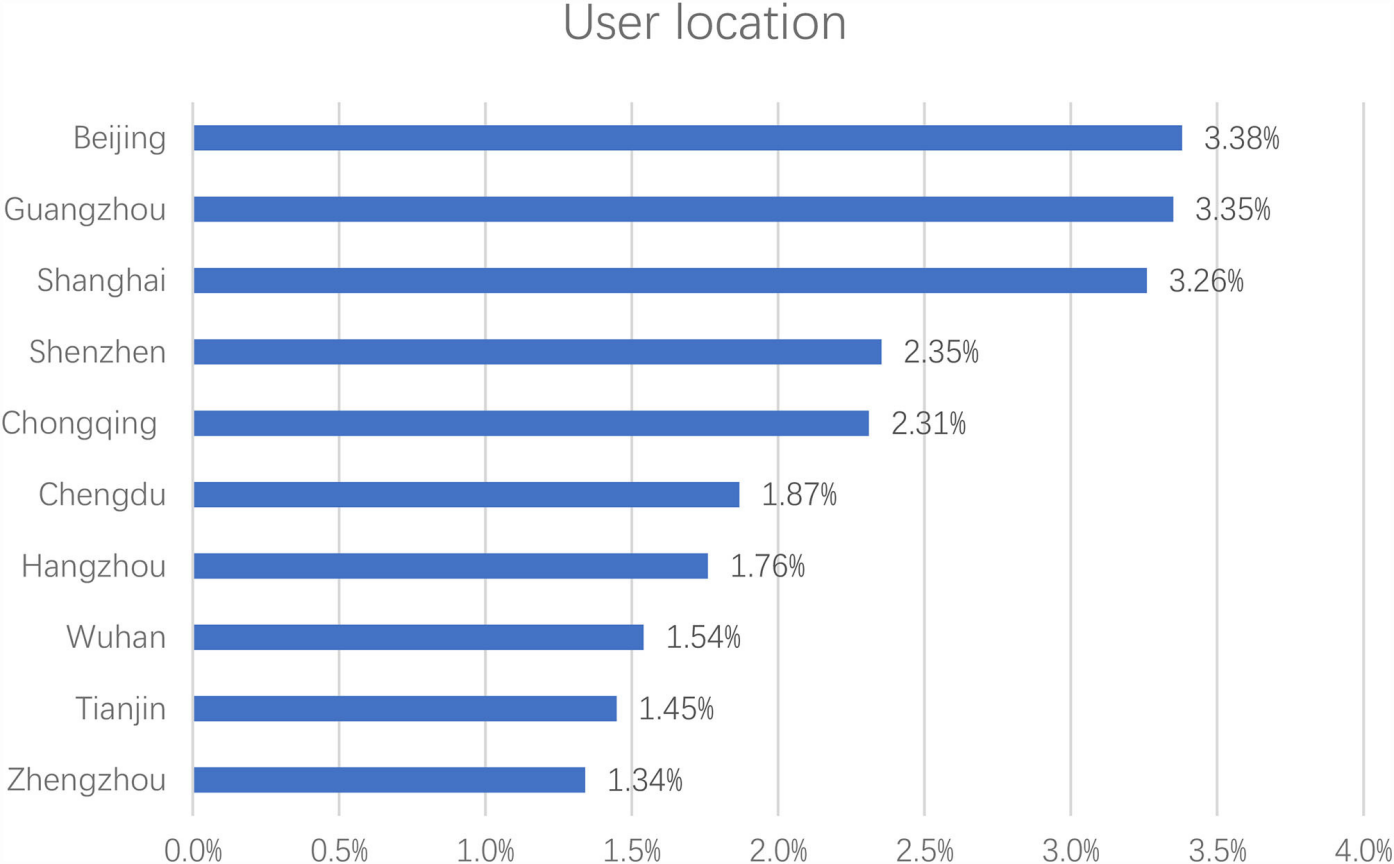
Top 10 location of the hashtag “#national mask campaign#” participants.

**Figure 7:**
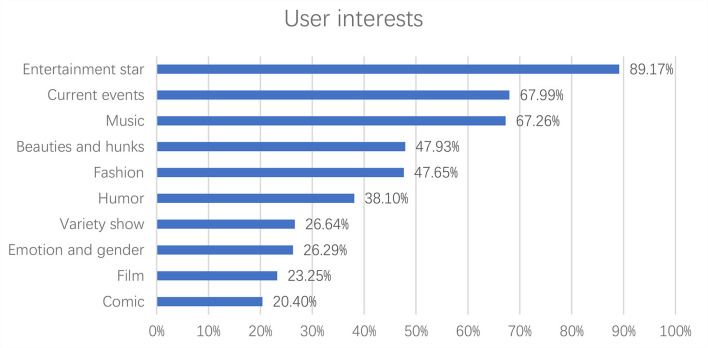
User interests of the hashtag “#national mask campaign#” participants.

Apparently, the Chinese celebrity idols played active roles in the hashtag health campaign “#national mask campaign#” on Sina Weibo, and their fans were mobilized and actively participated by reposting, commenting, and liking the hashtag posts. To investigate the characteristics of the participants of the hashtag health campaign, their social profile attributes were extracted and analyzed. The results in Figure 4 indicate that the average age of the participants was 22. As shown in Figure 5, there were more female participants (78.25%) than male participants (21.75%). As for location, most of the participants were from big cities, such as Beijing, Guangzhou, Shanghai, Shenzhen, Chongqing, Chengdu, Hangzhou, Wuhan, Tianjin, and Zhengzhou (refer to Figure 6). Figure 7 shows that entertainment stars comprised 89.17% of user interests of the hashtag participants, followed by current events (67.99%), music (67.26%), etc. Thus, hashtags play a key role as an indicator of collective identity as fandom publics. Based on the profiling analyses, the group profile of fandom publics hashtagging “#national mask campaign#” was depicted.

## Method Study 2

### Sample

In Study 2, an original nationwide survey and survey experiments were conducted to further investigate the fandom publics and the interrelationships among celebrities, fan identity, and collective action.

Because young people are the focus of this research, those aged from 14 to 35 were identified as the target population of the research, according to *The Middle- and Long-term Youth Development Plan (2016–2025)* released by the Central Committee of the Communist Party of China and the State Council. To recruit participants, InsightWorks, one of the professional Chinese survey companies, was used as the online panel provider, which comprised more than 4 million Chinese panel members. All the participants were Chinese citizens being aged between 14 and 35, recruited through a quota sampling process according to the education and gender distributions of the target population in the census. Participation in this study was voluntary, and participants were paid a standard fee for completing the questionnaire. A total of 2,211 participants completed the survey.

In the sample, participants were 14–35 years old (*M* = 23.96, *SD* = 6.38), and 51% were women, from 237 cities of 31 provincial units (excluding Hong Kong, Macao, and Taiwan). As for education background, 45.8% completed their Bachelor's education, 19.8% completed vocational education, 17% held a high school degree, 10.9% completed middle school education, 5.3% attended or completed graduate school, and 1.2% attended or completed primary school education. Among the participants, 54.6% were employed people, followed by 36.7% students, 6.6% freelancers, and 2.1% were unemployed. Regarding family economic status, 54.8% of the participants perceived their family economic condition as middle class, 38.2% as survived, 4.1% as poor, and 2.9% as rich.

### Measures

Fan identity was measured by asking the participants whether they have a celebrity idol (singers or actors) and fandom identification, including 5 items, including “I see myself as a fan of certain celebrity idol,” “Being a fan is important to me,” “I am a member of fan communities,” “I participate in fan activities online frequently,” and “I participate in fan activities offline frequently,” using a 5-point Likert scale ranging from 1 (strongly disagree) to 5 (strongly agree). The reliability alpha was 0.91 for this 5-item scale.

Civic participation was also measured. To compare the impact of celebrity idol and fandom community, different scenarios were provided in the survey. As for the do's and don'ts in public campaign narratives, including “bringing your own cutlery and using reusable shopping bags” and “not using plastic bags or not littering,” the participants were asked to indicate which one is more influential, “celebrity idol leading by example and appealing for more participation” or “advocacy from fandom community in the name of celebrity idol.” To further examine the degree of civic participation, regarding the scenario of “a natural disaster happened,” as for four types of civic engagement, i.e., “donating blood, money, or goods,” “voluntary activities,” “spreading the related information online *via* personal social media account,” and “persuading family members and friends to participate,” the participants were asked to indicate whether “celebrity idol leading by example and appealing for more participation” or “advocacy from fandom community in the name of celebrity idol” is more influential, or equally influential.

In addition, to further dig into the mechanism of fandom civic participation, a scenario experiment was set up. In the experiment, the participants were randomly assigned to a control group and two treatment groups. All the groups were told that recently, there is a severe earthquake in Country A, and thus, humanitarian assistance to the victims was also provided by the Chinese government, and an official app was released for collecting donations from the public. The participants were asked whether they would like to donate in this case. Different from the control group, Treatment Group 1 was told that their celebrity idol shared the information *via* his/her Weibo account and appealed for his/her fans to donate. Treatment Group 2 was told that not only their celebrity idol but also their fandom communities appealed for donation and added mentions (tags) to the celebrity idol's Weibo account in their posts with the donation receipt. We then compare whether with or without the influence from celebrity idol and influence from both celebrity idol and fandom communities, makes any difference on the civic participation. In this study, an earthquake being chosen as a scenario for the experiment was mainly due to two main considerations. First, during the COVID-19 pandemic, it was almost impossible to distinguish the influence of any campaign of mask wearing when most people in China had already realized its importance as a preventive measure. Second, according to WHO, with no warning at all, tens of thousands of people can be put in danger during an earthquake which can cause severe public health impacts and require emergency and humanitarian action (WHO, 2010, 2020c). Therefore, an earthquake rather than any infectious disease was used as a scenario for the experiment so as to minimize the emotional contagion from the ongoing pandemic that people were all experiencing.

## Results Study 2

### Fandom Publics Among Young Chinese

**Figure 8:**
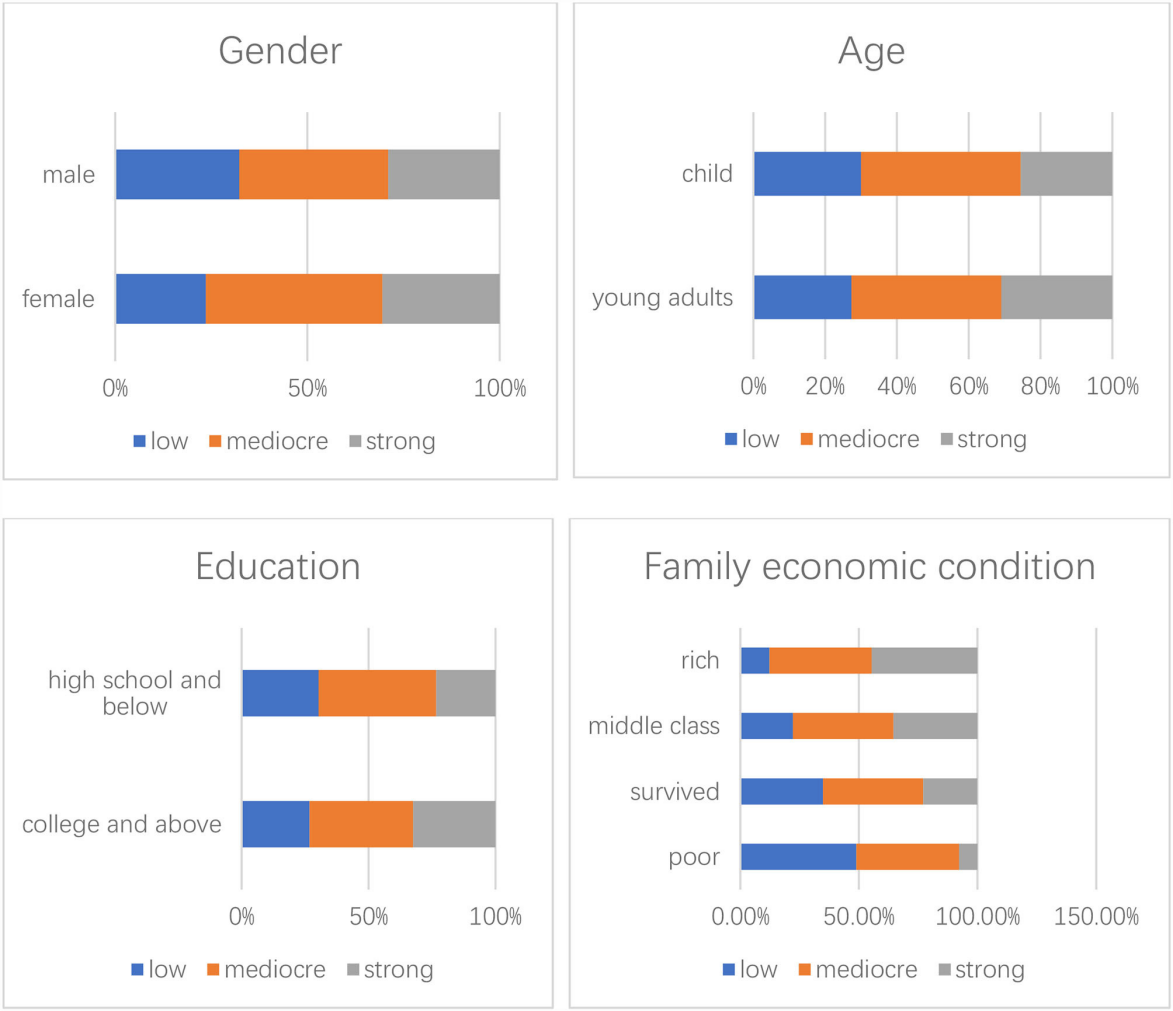
Profile of fandom publics among young Chinese.

Based on the nationwide survey, the majority (80.4%) had a celebrity idol. As for fandom identification, the responses originally measured by a 5-point Likert scale were recoded as 1 (weak), 2 (mediocre), and 3 (strong). Among the participants, 72.2% had a mediocre or strong level of feeling of fandom belonging. The results (see Figure 8) show fandom identification across gender, age, education, and family economic condition, which indicate that fan identity is a common phenomenon among young Chinese. According to the results, there are more women, while still a considerable number of men, self-identified as fandom members. Fandom identification is popular among both young adults and under age 18 populations, those high school educated below, and participants with college or above education and with different family economic conditions. These statistics demonstrate the profile of fandom publics among young Chinese.

### Influence of Celebrity Idol and Fandom Community in Different Scenarios and Different Types of Civic Engagement

**Figure 9:**
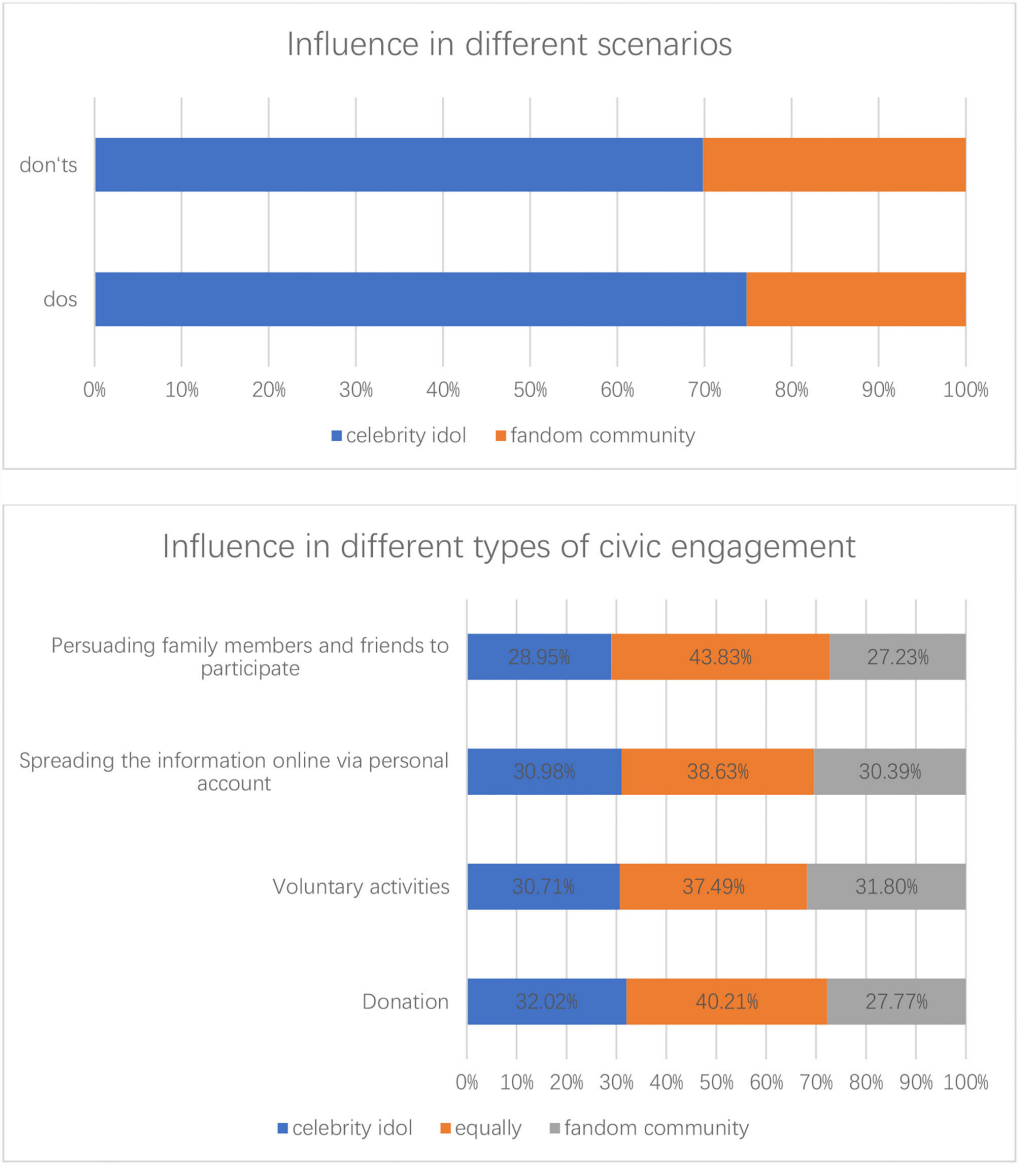
Influence of celebrity idol and fandom community in different scenarios and different types of civic engagement.

To compare the influence of celebrity idol and fandom community, both different scenarios and different types of civic engagement were investigated (see Figure 9). As for both do's and don'ts in public campaign narratives, “celebrity idol leading by example and appealing for more participation” was more influential than “advocacy from fandom community in the name of celebrity idol.” Regarding four different types of civic engagement, celebrity idol was slightly more influential in donation, spreading the related information online *via* personal social media account and persuading family members and friends to participate, while fandom community was a little more influential in voluntary activities, and a considerable proportion of the participants regarded celebrity idol and fandom community as equally influential in the four types of civic engagement.

### Mechanism of Fandom Civic Participation

Based on the experiment embedded in the nationwide survey, the results (refer to Table 2) show the mechanism of celebrity idol and fandom community in fandom civic participation. As Morgan and Rubin (2012) pointed out, simple randomization cannot guarantee data balance. To avoid the potential malfunction of randomization on the results, demographic and sociopolitical controls (such as gender, age, education, and occupation) were further added in the analysis of the experiment results. The results show that, in comparison with the control group, both celebrity idol and fandom community treatment groups have a positive trend on donation willingness, whereas only the effect of both celebrity idol and fandom community is statistically significant. In other words, in a charity donation scenario, despite an inconclusive effect of celebrity idol, the mobilizing power with celebrity idol and fandom community combined is noticeable.

**Table 2:** Experimental result of mobilizing effect.

	**Mobilizing donation**
Celebrity idol	0.250
	(0.177)
Celebrity idol and fandom	0.353^**^
	(0.169)
Num. Obs.	1777
AIC	1367.4
BIC	1427.7
Log.Lik.	−672.694
F	2.819

*The results controlled for gender, age, education, and occupation*.

*p < 0.1,

** *p < 0.05*,

***p < 0.01.

**Figure 10:**
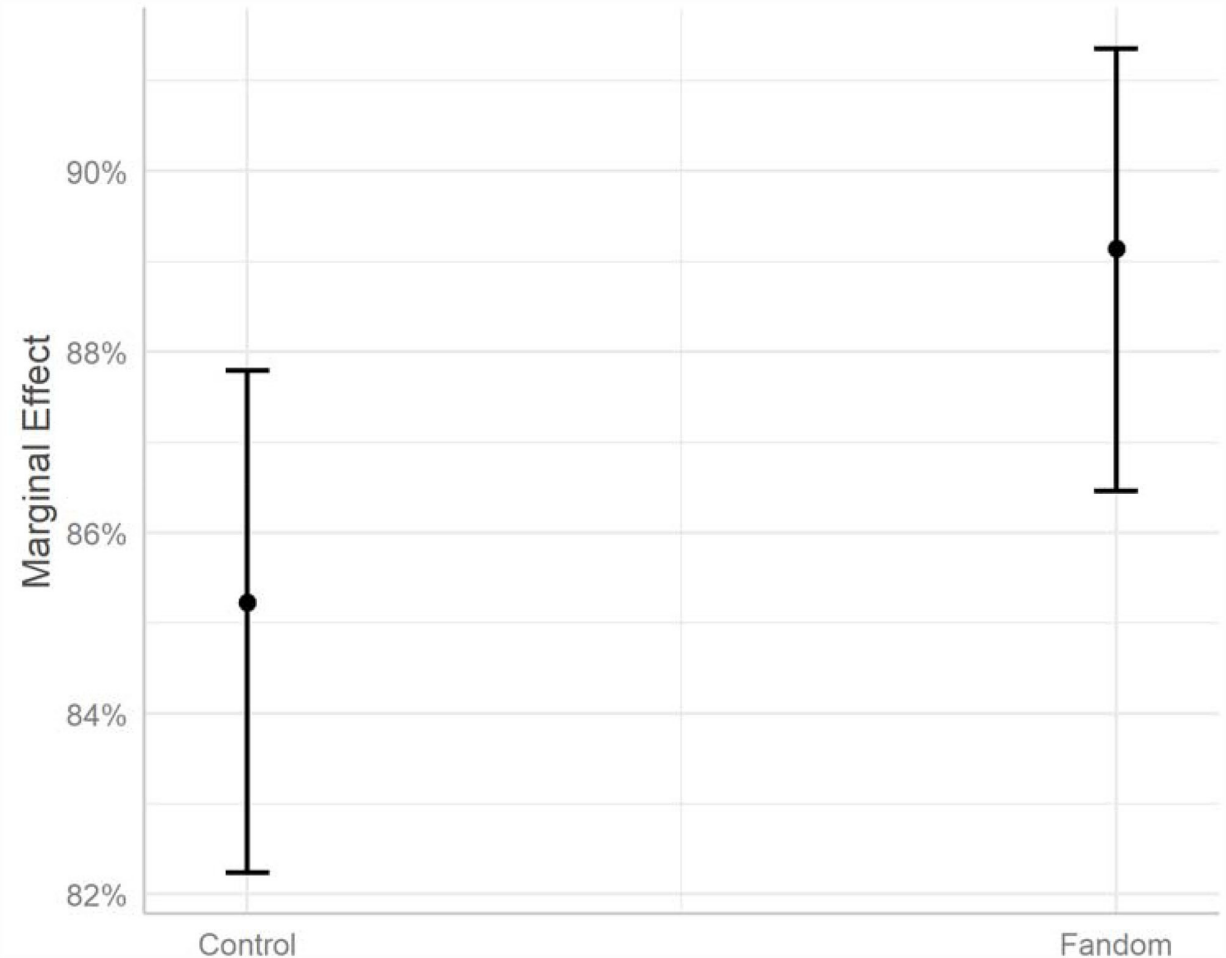
Effect of celebrity idol and fandom community combined on mobilizing donation.

As Figure 10 presented, the marginal effects of the celebrity idol and fandom community combined effect were further calculated. The proportion of being willing to donate was about 85%, given or taken the standard errors; the proportion in the celebrity idol and fandom community combined group is over 89%. It is worthy to note that the effects were examined with a very conservative design: participants in both control and treatment groups are likely to lean in the “willing to donate” option due to the social desirability or the experience of the COVID-19. Even in such cases, the combined effect of celebrity idol and fandom community stands out. One can expect the effect to be larger if the above factors are well-controlled.

## Discussion

With the rapid spread of the COVID-19 pandemic and multiple variants emerging around the world, the importance of prevention and intervention activities such as wearing face mask has been widely realized. During a special time of social distancing, quarantine, and isolation, digital health interventions have become more and more influential during the COVID-19 pandemic. Due to the affordances, social media can play a very important and active role in health campaigns and public engagement. Based on data collected on Sina Weibo and from a nationwide survey, this research identified the importance of hashtag mobilization and participation in the mask-wearing campaign during the COVID-19 pandemic, and the popularity of fandom publics and the mechanism of celebrity idol and fandom community were further examined with the aid of a nationwide survey.

During the escalation phase in the first half of 2020, the general population was recommended to wear face masks as a preventive intervention to slow the spread of COVID-19. Based on a systematic data search on Sina Weibo, the most popular microblogging platform, face mask became a hot word due to the mounting public concerns in the early stage of the COVID-19 pandemic. Related Weibo post of face mask increased sharply after the official interview of Dr. Zhong Nanshan was released on traditional mass media about the fact that COVID-19 can be spread from person-to-person. Due to the possible intermedia agenda-setting effects, millions of posts related to face mask indicated the power of CMC-supported collective action to raise public attention to face mask wearing as important prevention to slow and stop the spread of the virus.

By further investigating the mask-related posts, the findings show that hashtags became quite present as communication modalities for a social media health campaign. Topics posted on Sina Weibo have received millions of likes, comments, and reposts. Among various Weibo topics indicated by hashtags, “#national mask campaign#” topped the ranking, by comprising more than a quarter of the total discussion of the top 1,000 hashtag topics. Therefore, within the public discussion of interventions during the COVID-19 pandemic, hashtags show a strong impact on civic expression and participation on social media and emerged as meaningful tools for CMC-based health campaigns.

Taking the “#national mask campaign#” as a case study, this research further demonstrates the narrative agency in this social media health campaign (Yang, 2016). By hashtagging the “national mask campaign,” the participants not only expressed their own personal thoughts but also produced narratives collectively on social media. The findings of frequency analysis and word cloud analysis show that the coproduction of narratives emphasized prevention activities on the one hand and expressed solidarity in response to the COVID-19 pandemic on the other hand. As digital health interventions, the mask-wearing social media health campaign through hashtags attempted to both promote healthy behaviors and reduce psychological burden during the COVID-19 pandemic. Social media such as Sina Weibo can be very helpful for information sharing and the development of collective identity. Names of some celebrity idols were among those most frequently appeared words of the “#national mask campaign#,” indicating celebrities and their fans' active participation in this Weibo health campaign. As a social media health campaign, the “#national mask campaign#” was initiated by a Chinese celebrity, and those most influential posts with millions of reposts and hundreds of thousands of comments and likes were also from verified celebrity Weibo accounts. It is worthy to notice that the impact of celebrity idols and fandom communities becomes more present in civic engagement, including public health campaigns.

Apparently, the celebrity idols played active roles in the hashtag health campaign “#national mask campaign#” on Sina Weibo. Those influential posts by celebrity idols raised the considerable concern of mask wearing as one of the most effective public health measures and part of a comprehensive strategy of measures to prevent the spread of COVID-19. The celebrity idols acted as opinion leaders whose posts received more attention on social media. The fans of these celebrity idols were mobilized by the hashtag topic and actively participated by reposting, commenting, and liking the hashtag posts, so as to maintain its top position within the ranking. In this way, the hashtag topic was kept influential by the fandom and went beyond fandom to the more general public. By hashtagging, fandom publics expressed their connective identity and pursuit of shared goals (Mihailidis, 2020). As a specific type of media affordance, the hashtag also played a key role in the development of collective identity which was influential within this social media health campaign. Findings of the profiling analyses indicate the group profile of fandom publics hashtagging “#national mask campaign#” on Sina Weibo as young adults, more women, living in urban areas, with self-identified interests of entertainment stars, current events, and music, etc.

Based on a nationwide survey, this research investigated the popularity of fan identity and the mechanism of influence of celebrity idol and fandom community in civic engagement. Findings of the nationwide survey demonstrate fan identity as a common phenomenon among young Chinese across gender, age, education, and family economic condition, with 80.4% of the participants having a celebrity idol and 72.2% showing a mediocre or strong level of fandom identification. The results show that there are more women, while still a considerable number of men self-identified as fandom members. By examining different scenarios and different types of civic engagement, this research found that celebrity idol was regarded as more influential in both do's and don'ts public campaign narratives, while regarding four different types of civic engagement, i.e., donation, voluntary activities, spreading the related information online *via* personal social media account, and persuading family members and friends to participate, the influence of celebrity idol and fandom community varied slightly or was regarded as equally influential. The experiment embedded in the nationwide survey further disclosed the mechanism of fandom civic participation. The results show that in a charity donation scenario, both celebrity idol and fandom community have a positive trend on donation willingness whereas only the effect of both celebrity idol and fandom community combined is statistically significant. Thus, despite an inconclusive effect of celebrity idol influence, the mobilizing power with both celebrity idol and fandom community combined is noticeable.

## Conclusion and Limitations

During the global COVID-19 pandemic, social media showcase a great potential for public health campaigns and psychological support. Guided by the conceptual framework of media affordances, this research investigated the impact and mechanism of CMC, social identity, and collective action based on two consecutive empirical studies during the pandemic. By posting, liking, commenting, reposting, and hashtagging, young people in China shared information of interventions and expressed solidarity on a popular Chinese social media platform during the escalation phase in the first half of 2020. Universal masking is a preventive intervention to reduce transmission. Initiated by a celebrity, “#national mask campaign#” as hashtag mobilization and participation involved many celebrity idols and millions of fandom publics on Sina Weibo. The impact of narrative agency and coproduction of narratives was demonstrated through information sharing, connective identity formation, and collective action online and offline. With the popularity of fan identity and social media usage among young Chinese, celebrity idol and fandom community can play very influential roles in civic engagement and participation among the young in China. In the context of the global public health crisis, health information spreading can effectively stop the virus from spreading. Implications from this research point to a need for policy makers and public health professionals to pay more attention to social media to explore more possibilities for public health interventions. Public health social media campaigns, such as the hashtag “#national mask campaign#” on Sina Weibo, can help to make more people aware and know the importance of face mask wearing to stop the spread of COVID-19 and act accordingly. Initiated by celebrity idols as key opinion leaders, actively engaged by fandom communities, and gradually known by the general public, this national mask campaign can help people to wear mask together and be all right during the pandemic.

Although the findings of this research are interesting and important for social media health campaigns, limitations should also be noticed. Study 1 of this research is based on data collected *via* Sina Weibo posts, and thus, those deleted posts, if any, were not considered in this research. Focusing on fandom publics, the nationwide survey for Study 2 was conducted among young Chinese, and the scenarios designed and used were related to fans' experiences. Therefore, future studies can be conducted with the consideration of different social media, across diverse populations, regarding more scenarios, and in multiple sociocultural contexts.

## Data Availability Statement

The original contributions presented in the study are included in the article/supplementary material, further inquiries can be directed to the corresponding authors.

## Ethics Statement

Ethical review and approval was not required for the study on human participants in accordance with the local legislation and institutional requirements. Written informed consent from the participants' legal guardian/next of kin was not required to participate in this study in accordance with the national legislation and the institutional requirements. Written informed consent was not obtained from the individual(s) for the publication of any potentially identifiable images or data included in this article.

## Author Contributions

QJ designed the study and performed the data analysis and wrote the article. SL did the literature review. YH designed the experiments and analyzed the experiment data. JX designed the study and collected the Weibo data. All authors contributed to this study and approved the submitted version.

## Funding

This work was funded by a grant from the National Social Science Foundation of China (grant no. 20BXW127).

## Conflict of Interest

The authors declare that the research was conducted in the absence of any commercial or financial relationships that could be construed as a potential conflict of interest.

## Publisher's Note

All claims expressed in this article are solely those of the authors and do not necessarily represent those of their affiliated organizations, or those of the publisher, the editors and the reviewers. Any product that may be evaluated in this article, or claim that may be made by its manufacturer, is not guaranteed or endorsed by the publisher.
